# Perforated Appendicitis as a Herald of Appendiceal Adenocarcinoma: A Case Report and Literature Review

**DOI:** 10.7759/cureus.104587

**Published:** 2026-03-02

**Authors:** Saman Nekoobahr, Ricardo Olivas Lerma, Nawar Hakim, Jeffrey Sherwood

**Affiliations:** 1 Internal Medicine, Texas Tech University Health Sciences Center El Paso Paul L. Foster School of Medicine, El Paso, USA; 2 Internal Medicine, Texas Tech University Health Sciences Center El Paso, El Paso, USA; 3 Pathology, Texas Tech University Health Sciences Center El Paso, El Paso, USA; 4 Infectious Disease, Texas Tech University Health Sciences Center El Paso, El Paso, USA

**Keywords:** appendectomy, completion right hemicolectomy, incidental, mucinous appendiceal adenocarcinoma, perforated appendicitis

## Abstract

Appendiceal adenocarcinoma is a highly uncommon gastrointestinal tumor that can have severe consequences if not identified or properly treated. These malignancies are often diagnosed incidentally after appendectomy through histopathological evaluation of the appendix. Moderate- or poorly differentiated tumors frequently warrant completion right hemicolectomy followed by adjuvant chemotherapy. We present the case of a 76-year-old male with a history of hypertension, benign prostatic hyperplasia, and seborrheic keratosis who was admitted for management of a ruptured appendix associated with an intra-abdominal abscess. He improved symptomatically after undergoing CT-guided abscess drainage and receiving antibiotics targeting bowel flora. However, a formal diagnosis of appendiceal cancer was established when histopathology from interval appendectomy seven weeks later revealed invasive adenocarcinoma with extensive mucin production invading through the appendiceal wall and obstructing the distal appendix. He subsequently underwent completion right hemicolectomy, with histopathology demonstrating no lymphatic invasion and clear surgical margins. At the time of this report, he was preparing to begin a 5-fluorouracil-based chemotherapy regimen. This case highlights perforated appendicitis as a potential harbinger of appendiceal carcinoma and reviews the diagnostic and therapeutic considerations for mucinous appendiceal adenocarcinomas, emphasizing the importance of recognizing malignancy and intervening promptly in the context of perforated, acute appendicitis.

## Introduction

Appendiceal adenocarcinoma is a rare gastrointestinal neoplasm originating from the appendix, affecting approximately one to two individuals per million annually in the United States [1]. These malignancies may be diagnosed during an acute episode of appendicitis in up to 50% of cases, although their rarity and nonspecific symptoms often complicate early detection [2,3]. Clinical manifestations can include generalized abdominal pain, ascites, or a palpable abdominal mass. Given the high propensity for perforation, frank sepsis is also possible. In one study by Cerame, appendiceal adenocarcinoma was found to perforate in as many as 55% of patients, making it the most frequently perforating carcinoma of the gastrointestinal tract. Preoperative diagnosis was exceedingly rare, with only three cases identified in that review, typically during evaluation for primary malignancy elsewhere in the colon. Additionally, incidental or postmortem diagnosis occurred in approximately 8% of cases [4]. Despite its rarity, early detection is critical due to the high metastatic potential and poor prognosis of these tumors.

Appendiceal adenocarcinomas are classified into three histologic subtypes: mucinous, colonic, and signet-ring cell. The patient in this report had the mucinous subtype, which is typically graded as well, moderately, or poorly differentiated. Overall, appendiceal adenocarcinomas share phenotypic characteristics with colonic adenocarcinomas [5,6]. However, mucinous adenocarcinomas of the colorectum exhibit a distinct mutation profile compared with non-mucinous tumors, potentially linked to KRAS mutations. A relationship has also been observed between mucinous tumors of the appendix and the ovary, as both tumor types demonstrate higher rates of KRAS mutations than other carcinomas in these organs [5,7-10]. Further genomic analysis of 105 genes commonly implicated in appendiceal cancer has shown that tumor suppressor inactivation (e.g., TP53 and SMAD4) combined with activation of oncogenes such as KRAS, GNAS, and BRAF is necessary for tumorigenesis [11].

Staging of appendiceal adenocarcinomas follows the American Joint Committee on Cancer (AJCC) ninth edition tumor-node-metastasis system. T represents the size and extent of the primary tumor, N indicates regional lymph node involvement, and M denotes distant metastasis. In this case, the patient’s tumor was classified as pT3NXM1, indicating invasion through the muscularis propria into the subserosa or mesoappendix with distal appendiceal destruction, no regional lymph nodes submitted or identified, and microscopic confirmation of distant metastasis. Had the mucinous tumor ruptured and disseminated intraperitoneally, it would be classified as pseudomyxoma peritonei, characterized by mucin-producing cells seeding the peritoneum and producing the classic “jelly belly” appearance [12].

Management of mucinous appendiceal adenocarcinoma depends on histologic differentiation. Localized, well-differentiated (G1) tumors that are completely resected via appendectomy may be observed due to low lymph node involvement. Moderately (G2) or poorly differentiated (G3) localized tumors typically warrant completion right hemicolectomy followed by adjuvant chemotherapy due to higher lymphatic spread risk. Common regimens include a three- to six-month course of 5-fluorouracil (5-FU) with oxaliplatin. Asare et al. demonstrated that adjuvant chemotherapy significantly improves survival in both mucinous (HR for death 0.79, 95% CI 0.69-0.90) and non-mucinous tumors (HR 0.84, 95% CI 0.75-0.95) [13].

## Case presentation

A 76-year-old male with a past medical history of hypertension, benign prostatic hypertrophy, and seborrheic keratosis presented to the emergency department with several days of worsening diffuse abdominal pain, accompanied by nausea and subjective fevers and chills. On arrival, he was afebrile and hemodynamically stable.

Initial laboratory studies revealed a mildly elevated peripheral white blood cell count of 12.3 × 10⁹/L, with 66% segmented neutrophils, suggestive of an acute inflammatory or infectious process. Renal function and liver-associated enzymes were within normal limits, and blood cultures were sterile. Detailed admission laboratory results are summarized in Table 1.

**Table 1:** Laboratory findings on admission ALT, alanine aminotransferase; AST, aspartate aminotransferase; BUN, blood urea nitrogen

Laboratory test	Patient value	Normal range	Comment
White blood cell count	12.3 × 10⁹/L	4.0-10.0 × 10⁹/L	Elevated
Neutrophils (segmented)	66%	40-75%	Normal
Hemoglobin	14.4 g/dL	12-16 g/dL	Normal
Platelet count	320 × 10⁹/L	150-400 × 10⁹/L	Normal
Blood glucose	99 mg/dL	70-110 mg/dL	Normal
Renal function (BUN/creatinine)	Normal	Normal	-
Liver function tests (ALT, AST, and bilirubin)	Normal	Normal	-

**Figure 1:**
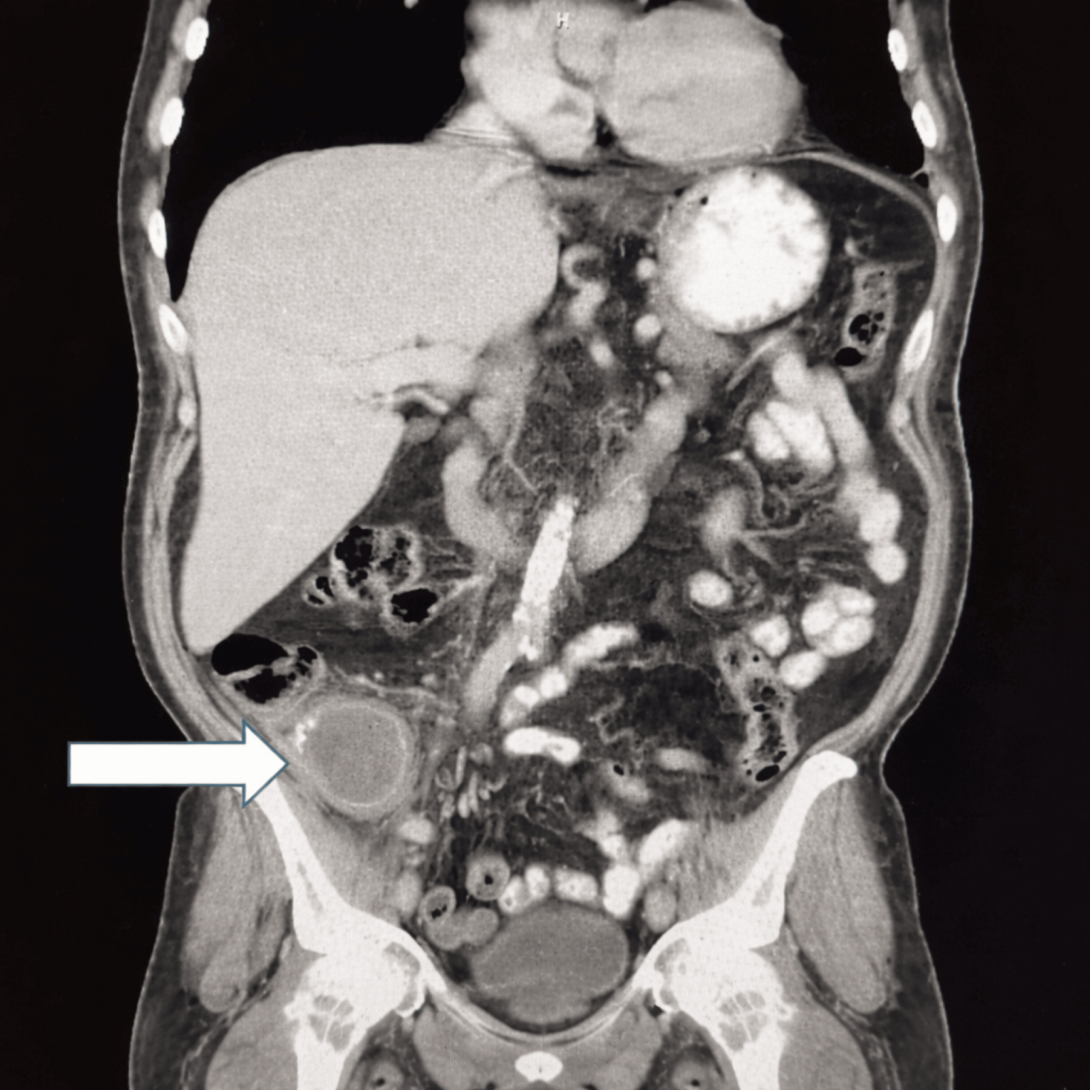
CT of the abdomen and pelvis with contrast showing appendiceal perforation and a rim-enhancing abscess in the right lower quadrant (arrow)

The patient underwent a contrast-enhanced CT scan of the abdomen and pelvis, which revealed no evidence of obstruction or abnormal bowel wall thickening. However, a perforated appendix with a right lower quadrant abscess measuring 6.9 × 5.4 × 5.0 cm was identified (Figure 1).

He was started on broad-spectrum antibiotics with piperacillin/tazobactam. General Surgery was consulted and recommended CT-guided drain placement as a temporary measure to bridge him to an interval appendectomy. Twenty milliliters of thick, purulent fluid were aspirated and sent for culture. Fluid culture grew *Escherichia coli* resistant to amoxicillin/clavulanate but susceptible to all other reported antibiotics. The patient was transitioned to oral ciprofloxacin in combination with Augmentin and discharged with a plan to complete a two-week course of post-drainage antibiotics.

A repeat abdominal CT two weeks later showed a slight reduction in abscess size to 2.9 × 1.8 cm, compared with 3.8 × 2.4 cm previously. The drain was found to no longer communicate with the small residual collection, resulting in minimal output, and was subsequently removed. The patient received an additional two-week course of Augmentin and ciprofloxacin.

**Figure 2:**
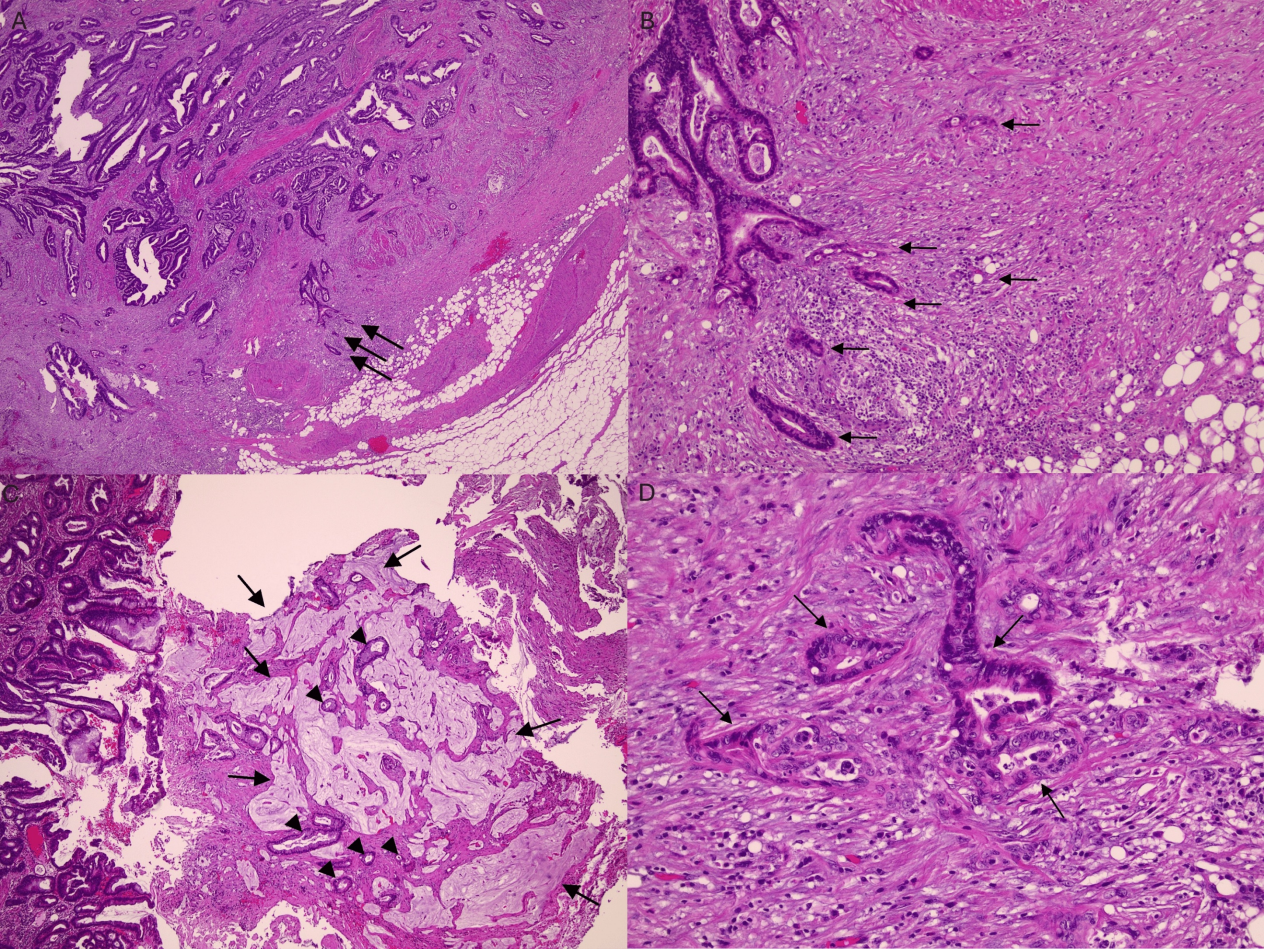
Histopathology of the appendix (A) Low-power view showing infiltrating adenocarcinoma involving the entire appendiceal wall and invading peri-appendiceal tissue. Arrows indicate tumor glands in peri-appendiceal tissue (H&E stain, ×20). (B) Tumor invading peri-appendiceal tissue with desmoplastic response. Arrows indicate invasive adenocarcinoma glands (H&E stain, ×100). (C) Tumor with focal extensive mucin production; pools of mucin contain occasional malignant glands (arrows/arrowheads) (H&E stain, ×40). (D) High-power view showing malignant glands lined by cells with nuclear overlap and stratification, featuring vesicular, hyperchromatic nuclei with prominent nucleoli (H&E stain, ×200). Pathology slides and review courtesy of Nawar Hakim

Seven weeks later, he was readmitted from the general surgery clinic for a scheduled interval robot-assisted laparoscopic appendectomy. Intraoperatively, the left appendiceal stump was encapsulated, and mucin was grossly observed. The stump appeared indurated and inflamed, but no gross purulence was noted. The appendix was resected and sent for histopathological evaluation, which revealed an invasive appendiceal adenocarcinoma, moderately differentiated (G2), pT3NXM1, measuring 2.0 × 1.0 × 1.0 cm. Extensive mucin production was present, with invasion through the full thickness of the appendiceal wall and obstruction of the distal appendix (Figure 2).

The tumor diffusely involved the appendix and its base. Surgical resection margins also showed involvement with destruction of the distal appendix. With this information, the patient promptly consented to a robot-assisted laparoscopic right hemicolectomy with primary anastomosis. Surgical pathology from this procedure revealed a single focus of acellular mucin near the peri-colonic adipose tissue at the previous appendectomy site. Twenty lymph nodes were negative for metastatic carcinoma, and all resection margins were free of dysplasia, malignancy, or acellular mucin. A port-a-cath was placed in anticipation of initiating adjuvant 5-FU-based chemotherapy. Notably, the patient’s staging carcinoembryonic antigen (CEA), carbohydrate antigen 19-9 (CA19-9), and cancer antigen 125 (CA125) were all within normal limits. Similarly, staging CT imaging of the chest, abdomen, and pelvis showed no evidence of metastatic lesions.

## Discussion

Tumors of the appendix account for less than 0.5% of all gastrointestinal tumors and are found in only approximately 1% of appendectomy specimens [14]. With a growing body of literature suggesting that short-term medical management of uncomplicated appendicitis may involve antibiotics alone, these neoplasms must be considered, particularly in patients with cancer-related risk factors such as advanced age or complications like perforation [15,16].

Medical management of moderately or poorly differentiated mucinous appendiceal adenocarcinoma typically involves completion right hemicolectomy followed by adjuvant chemotherapy due to higher rates of lymph node involvement. Conversely, localized, well-differentiated mucinous appendiceal adenocarcinomas that are completely resected by appendectomy are often observed, as lymph node involvement is rare and right hemicolectomy does not confer a survival benefit.

Surgical management of ruptured appendiceal mucinous lesions should generally be limited to appendectomy or right hemicolectomy (if the rupture is contained by the right colon and mesentery), peritoneal washing with fluid cytology, careful inspection of the abdominal cavity with documentation, and biopsy of any suspicious peritoneal lesions. It is also essential to thoroughly irrigate the abdomen and surgical wounds to minimize implantation of tumor cells. Advanced cytoreductive surgery should be reserved for surgeons with extensive experience in peritoneal malignancies.

A study by Lamarca et al. demonstrated that patients with appendiceal neoplasms presenting with acute appendicitis were more likely to have appendiceal perforation (80%), whereas those presenting with symptoms other than appendicitis had a perforation rate of only 20%. This study established a direct relationship between appendiceal perforation and stage at diagnosis, with earlier stages observed in patients with perforation (χ² = 14.9; p < 0.001) [17].

Recent data have further clarified the prognostic importance of mucin cellularity in appendiceal mucinous neoplasms. Patients with acellular peritoneal mucin (AJCC M1a) demonstrate significantly improved outcomes compared with those whose peritoneal deposits contain neoplastic epithelium (cellular mucin, M1b). In a retrospective cohort of 164 patients undergoing cytoreductive surgery and HIPEC, individuals with acellular mucin experienced no recurrences and only one death over a median follow-up of 7.6 years, whereas those with cellular mucin had a 66% recurrence rate and a five-year recurrence-free survival of approximately 40.5% [18]. These findings underscore the importance of detailed pathologic assessment of mucin deposits, as cellularity directly influences staging, recurrence risk, and long-term survival.

Despite conflicting evidence, medical oncologists often extrapolate data from node-positive colon cancer, highlighting the efficacy of adjuvant 5-FU and oxaliplatin-based chemotherapy, particularly for patients with non-mucinous appendiceal adenocarcinomas [19]. Our patient is planning to begin the standard six-month course of 5-FU.

Although serum tumor markers are commonly used to assess prognosis and monitor treatment response in various malignancies, their utility in appendiceal cancer is less certain. In one retrospective cohort of 1,338 patients with appendiceal cancer, 1,080 (80%) had evidence of metastatic disease at diagnosis. CEA, CA19-9, and CA125 were elevated in 742 (56%), 381 (34%), and 312 (27%) of patients, respectively. Patients within the top 10th percentile of marker elevation had significantly worse survival [20]. From this standpoint, our patient may have been fortunate to have normal tumor marker levels and no overt evidence of metastatic disease during staging.

Lastly, it is noteworthy that our patient reported a history of multiple seborrheic keratosis lesions over a seven-year period, predominantly affecting his upper torso. While this could raise suspicion for the Leser-Trélat sign, seborrheic keratosis is common in older adults, and a rapid onset of lesions over three to six months is more characteristic of underlying malignancy [21].

## Conclusions

Our patient’s case underscores the importance of recognizing and managing mucinous appendiceal adenocarcinoma. Such neoplasms should be considered in elderly patients presenting with acute appendicitis, particularly in the setting of perforation. Despite their rarity, timely diagnosis and appropriate surgical intervention are critical both to resolve the acute inflammatory process and to reduce the risk of metastatic spread. While ongoing studies may provide more data on the medical management of mucinous appendiceal adenocarcinoma, surgical management remains the cornerstone of treatment. Finally, further research into effective screening tools for these malignancies would be valuable, given that most cases are difficult to diagnose preoperatively and are often identified incidentally.
